# Prävention und Management von postinterventioneller Gewichtszunahme

**DOI:** 10.1007/s00508-023-02273-6

**Published:** 2023-10-11

**Authors:** Renate Kruschitz, Markus Fahrnberger, Daniel Moritz Felsenreich, Claudia Ress, Barbara Andersen, Kadriye Aydinkoc-Tuzcu, Christian Ciardi, Simone Leonore Huber, Florian W. Kiefer

**Affiliations:** 1 Abteilung für Innere Medizin, Krankenhaus der Elisabethinen, Klagenfurt, Österreich; 2 Sonderkrankenanstalt Rehabilitationszentrum Alland, Alland, Österreich; 3 Klinische Abteilung für Viszeralchirurgie, Universitätsklinik für Allgemeinchirurgie, Medizinische Universität Wien, Wien, Österreich; 4 Universitätsklinik für Innere Medizin I, Medizinische Universität Innsbruck, Innsbruck, Österreich; 5 Psychologische Praxis psychologie-andersen.at, Wien, Österreich; 6 5. Medizinische Abteilung für Endokrinologie, Rheumatologie und Akutgeriatrie, Klinik Ottakring, Wien, Österreich; 7 Abteilung für Innere Medizin, Krankenhaus St. Vinzenz, Zams, Österreich; 8 1. Medizinische Abteilung mit Diabetologie, Endokrinologie und Nephrologie, Karl Landsteiner Institut für Adipositas und Stoffwechselerkrankungen, Klinik Landstraße, Wien, Österreich; 9 Klinische Abteilung für Endokrinologie und Stoffwechsel, Universitätsklinik für Innere Medizin III, Medizinische Universität Wien, Währinger Gürtel 18–20, 1090 Wien, Österreich

**Keywords:** Adipositas, Metabolische Adaptierung, Lebensstil, Energieverbrauch, Hunger und Sättigung, Obesity, Metabolic adaptation, Lifestyle, Energy expenditure, Hunger and satiety

## Abstract

Die Ursachen der postinterventionellen Gewichtszunahme nach Lebensstiländerung, psychologischer Therapie, Pharmakotherapie oder chirurgischen Maßnahmen gehen weit über einen Motivations- oder Compliance-Verlust der Betroffenen hinaus. Der Gewichtszunahme liegen komplexe periphere und zentrale Mechanismen zugrunde, deren Ausmaß individuell unterschiedlich zu sein scheint und die darauf ausgerichtet sind, die Nahrungszufuhr durch reduziertes Sättigungs- und vermehrtes Hungergefühl zu erhöhen (gastrointestinale Hormone) und den Energieverbrauch zu reduzieren (metabolische Adaptierung). Diese Mechanismen erschweren das Abnehmen und Gewichthalten in einem „adipogenen“ Lebensraum, wie wir in weltweit immer häufiger vorfinden, ungemein. Das Verständnis dieser molekularen Mechanismen sollte in die Planung von Therapieprogrammen zur langfristigen Gewichtsreduktion, welche eine entsprechende Nachsorge zur Prävention und individualisierten Therapie einer postinterventionellen Gewichtszunahme beinhalten sollten, miteinbezogen werden. Dabei empfiehlt es sich, die therapeutischen Maßnahmen und Kontrollintervalle nach dem Ausmaß der Gewichtszunahme pro Zeitintervall auszurichten.

## Abstract (en)

Decreasing levels of patient motivation or compliance are far from being the only causes of postinterventional weight regain after lifestyle, psychological, pharmacological and surgical interventions. Weight regain originates from a complex and individually varying set of central and peripheral mechanisms, with the overall purpose of increasing food intake by both stimulating hunger and reducing satiety (mediated by gastrointestinal hormones) and decreasing the body’s energy demands (via metabolic adaption). These mechanisms counteract any attempts to reduce or maintain body weight in today’s increasingly prevalent adipogenic environments. The knowledge about the biological mechanisms of body weight regulation should be taken into consideration when planning treatment programs for long-term weight reduction, including follow-up treatment for the prevention and individualized treatment of postinterventional weight regain. Therapeutic measures as well as the frequency of medical follow-ups should be based on the extent of weight regain.

Als postinterventionelle Gewichtszunahme („weight regain“) bezeichnet man jenen Prozess, im Zuge dessen es nach einer Maßnahme zur Gewichtsreduktion zu einer neuerlichen Gewichtszunahme kommt. Eine Gewichtszunahme nach einer Intervention (Lebensstiländerung, psychologische Therapie, Pharmakotherapie oder chirurgische Maßnahmen) ist häufig. Sie beruht auf hormonellen und metabolischen Veränderungen, Wiederauftreten von gewichtsfördernden Verhaltensmustern, fehlender Therapieadhärenz, psychosozialen Problemen, psychischen Erkrankungen und anatomischen Adaptierungen nach operativen Eingriffen zur Gewichtsreduktion (Tab. 1; [1]). Maßnahmen zur Vermeidung und Behandlung einer postinterventionellen Gewichtszunahme (Tab. 2) sollten ein grundlegender Bestandteil jeglicher Adipositastherapie sein [2, 3].

**Table 1:** 

**1.1. Metabolische und therapeutische Faktoren**
Veränderte Medikation, gewichtsfördernde Medikamente: – Antidepressiva: Mirtazapin, Paroxetin, Amitriptylin, Nortriptylin – Antipsychotika: Lithium – Neuroleptika: Clozapin, Olanzapin – Antidiabetika: Insulin, Sulfonylharnstoffe, Glitazone – Antihypertensiva: Betablocker – Kontrazeptiva: Gestagene – Kortikosteroide
Vorliegen einer Endokrinopathie: – Hypercortisolismus – Schilddrüsenfunktionsstörung – Androgene Tumoren – Polyzystisches Ovarsyndrom
Genetische Syndrome (z. B. Prader-Willi-Syndrom) bei Auftreten von Adipositas bereits im Kindesalter
Niedriger Anteil an aktiver Körperzellmasse (BCM)
Vermehrter Hunger, verminderte Sättigung
**1.2. Verhaltens- und Umgebungsfaktoren**
Zu geringes Bewegungspensum, -angebot
Sozioökonomische Grundlagen
**1.3. Psychologische Faktoren** [24–30]
Psychische Erkrankungen: – Essstörungen – Depressionen – Persönlichkeitsstörungen (z. B. Borderline-Syndrom) – Angststörungen – Suchterkrankungen – Schizophrenie – Traumatische Erlebnisse
Substanzgebundene (Nahrungsmittel) und -ungebundene (Essverhalten) Suchtsymptomatik, u. a. chemisches und emotionales Craving, Kontrollverlust, Rückfallmanagement
Krankhafte Essmuster (s. unten)
Soziokulturelle Grundlagen (z. B. Biografie, Lerngeschichte, Kultur, Wirtschaft)
Über die Energiezufuhr hinausgehende Funktionen des Essens (u. a. Belohnungs- und Verstärkersystem, Entspannung, Langeweile, Stressabbau, unbewusstes Essen, mangelnde Impulskontrolle)
Selbstbild, Selbstwert
**1.4. Diätetische Faktoren**
Zu hohe Energiezufuhr
Krankhafte Essmuster: – Essattacken (Binge Eating) – Permanentes „Dahinessen“ (Grazing) – Essen in der Nacht (Night-Eating-Syndrom) – Primäre Aufnahme von weichen Speisen (Soft-Food-Syndrom) – Vermeidendes Essverhalten nach bariatrischer Chirurgie („Postsurgical Eating Avoidance Disorder“) – Andere Essstörungen
**1.5. Anatomische und chirurgische Faktoren nach bariatrischer Operation**
*Dilatation der gastrojejunalen Anastomose:* Durch Erweiterung der Anastomose zwischen Magenpouch und Jejunum kann es zu einer schnelleren Pouchentleerung und somit verminderten Restriktion kommen. Neben einer Gewichtszunahme kann dies auch zu Dumpingsymptomatik, besonders bei Nahrungsaufnahme nichtkomplexer Kohlenhydrate, führen [31]
*Dilatation des Magenpouches:* Besonders bei High-Volume-Eatern, die trotz Sättigungsgefühl weiter essen, kann es zu einer Vergrößerung des Fassungsvolumens des Pouches, verminderten Restriktion und somit Zunahme der möglichen Portionsgrößen kommen [32, 33]
*Vorhandensein einer gastrogastralen Fistel:* Besonders bei PatientInnen mit chronischen Ulzera kann eine Verbindung zwischen Pouch und Restmagen entstehen. Dadurch kommen sowohl der Restmagen als auch der ausgeschaltete biliopankreatische Schenkel wieder in die Nahrungspassage [34]
*Fundusrest:* Bleibt bei der primären Operation ein Teil des Fundus am Pouch bestehen, so kann es nach initialer Gewichtsabnahme wieder zu einer Gewichtszunahme kommen [32, 35]
*Dünndarmadaptation:* Wenige PatientInnen leiden trotz einwandfreien Pouches unter einer Gewichtszunahme. Dies kann auf eine Adaption des in der Essenspassage verbliebenen Dünndarms, der die Nahrungsaufnahme des aus der Passage genommenen Dünndarms ausgleicht, zurückgeführt werden [32, 36]

**Table 2:** 

**2.1. Medizinische, diätologische, sportmedizinische und psychologische Kontrolltermine**
Kontrolle der Umsetzung von diätetischen Empfehlungen
Überprüfung des aktuellen Ausmaßes und der Arten der Bewegung (s. Tab. 2, Maßnahmen 2.4)
Evaluierung psychosozialer Veränderung (privates und berufliches soziales Umfeld, subjektive Erwartungen und Bedürfnisse etc.)
Evaluierung hinsichtlich des Auftretens von Depressionen, Suchtverschiebung und anderer psychischer Erkrankungen
Entwicklung von krankhaftem Essverhalten (s. Tab. 1, Faktoren 1.4)
**2.2. Eigenbeobachtung**
Führung eines Ernährungs‑, Bewegungstagebuches [2]
Verwendung eines Schrittzählers, Aktivitätstrackers [21, 37]
Regelmäßige Gewichtskontrollen (1- bis 2‑mal/Woche) [2]
Bewusstmachung/Stärkung der Wahrnehmung von Essverhalten und Umgang damit (s. Tab. 2, Maßnahmen 2.5)
**2.3. Diätetische Maßnahmen**
Individuell angepasste Empfehlungen, die den Energie- und Eiweißbedarf, die Vorlieben, Unverträglichkeiten, Vortherapien (konservativ, bariatrisch) und Begleiterkrankungen der PatientInnen berücksichtigen [21]
Exploration von möglichen Ernährungsdefiziten
Exploration folgender Faktoren: – Aktuelle Ernährungs- und Trinkgewohnheiten (cave: Softdrinks, Alkohol) – Aufklärung/Erlernen der Unterscheidung zwischen Durstgefühl und Hunger – Umgang mit Heißhunger/Gusto/Snacking – Planung von regelmäßigen Mahlzeiten, Mahlzeitenabständen – Bewusstes Essen mit Wahrnehmen von Essgeschwindigkeit und Kaufrequenz
Intermittierender Einsatz von Formuladiäten zur Gewichtsstabilisierung [23]
**2.4. Bewegungsmaßnahmen **[20, 38, 39]
Bewegung mit mittlerer Intensität im Ausmaß von mindestens 150 min, besser 250–300 min pro Woche; dies entspricht etwa einem zusätzlichen Energieverbrauch von 2000 kcal pro Woche. Zusätzlich mindestens 2 Einheiten mit muskelkräftigender Bewegung (je mindesten 10 min)
Reduktion der sitzenden Tätigkeiten
Erhöhung der Alltagsaktivität
**2.5. Psychologische Maßnahmen **[40]
Eingeübte Verhaltensänderungsstrategien [41] und Zielorientierung [42] evaluieren
Reflexion von individuellem Emotions- und Stressmanagement [42] sowie Evaluierung der Copingstrategien (u. a. Entspannungstraining, Achtsamkeitsübungen) und der Rückfallprophylaxe
Intrinsische Motivation [24], Selbstführsorge und Selbstwirksamkeit fördern und stärken
Spezielle Aspekte, die bei PatientInnen nach bariatrischer Operation besonders berücksichtigt werden sollten: – Jegliche Psychopathologie kann das Outcome beeinflussen [43–45] – Abklärung der Suizidalität [44, 46–48] – Suchtverlagerung [36], Substanzmissbrauch und selbstschädigendes Verhalten [43, 46–50] – Förderung des positiven Einflusses von psychosozialen Interventionen [48] – Abklärung traumatischer Erlebnisse [25] – Längerfristige postoperative Begleitung begünstigt den Erhalt der Gewichtsreduktion [51]
Bei Vorhandensein von psychischen Erkrankungen und/oder psychopharmakologischer Medikation empfiehlt sich bereits präinterventionell und insbesondere bei Auftreten einer postinterventionellen Gewichtszunahme und/oder Verschlechterung des psychischen Befindens eine rasche Kontaktaufnahme mit der/dem behandelnden PsychologIn und FachärztIn
Exploration psychischer Befindlichkeiten und klinischer Symptome (Entwicklung von psychischen Störungen, Suchtverlagerung hin zu substanzgebundenen und -ungebundenen Süchten) [52]
Auffälligkeiten im Ess- und Trinkverhalten, die eine Gewichtszunahme begünstigen, abklären und bearbeiten (u. a. Craving, Grazing, Nibbling, Loss of Control Eating, Overeating, Emotional Eating, Chewing and Spitting, Night-Eating-Syndrom)
**2.6. Medizinische Maßnahmen**
Anpassung der Medikation an den Therapieverlauf und Begleiterkrankungen
Fortführung bzw. Start mit einer antiadipösen Pharmakotherapie (GLP-1 Rezeptoragonisten (zukünftig GIP/GLP-1 Rezeptoragonisten), Naltrexon/Bupropion, Lipasehemmer) insbesondere bei einer moderaten bzw. raschen klinisch relevanten Gewichtszunahme (s. Abschnitt „Beurteilung einer relevanten postinterventionellen Gewichtszunahme“) [21, 22]
Exploration von möglichen Mikro- und Makronährstoffdefiziten
Internistische Evaluierung bei atypischem Therapieverlauf bzw. unklarer Gewichtszunahme, ggf. erneute endokrinologische Diagnostik bei klinischen/laborchemischen Auffälligkeiten
Evaluierung der Möglichkeit für einen Rehabilitationsaufenthalt mit Stoffwechselschwerpunkt
**2.7. Chirurgische Maßnahmen**
*Die Art der Reversionsoperation sollte im Kontext mit den Ursachen der Gewichtszunahme stehen *[1]:
Pouchresizing [32, 33]
Pouchbanding [53]
Pouchresizing + Pouchbanding, um Redilatation zu verhindern [32]
Neuanlage der gastrojejunalen Anastomose; endoskopische Verengung der gastrojejunalen Anastomose [54]
Versetzen der Fußpunktanastomose (Verlängerung des komplett aus der Essenspassage genommenen biliopankreatischen Schenkels) [36]
Umwandlungsoperation: Bei PatientInnen mit „sleeve gastrectomy“ kann bei klinisch relevanter Gewichtszunahme eine Umwandlung auf Y‑Roux-Magenbypass oder „single anastomosis duodeno-ileal bypass + sleeve gastrectomy“ (SADI-S) erfolgen, um eine Malabsorption hinzuzufügen [55]

## Physiologische Grundlagen der Gewichtszunahme

### Gastrointestinale Hormone

Gastrointestinale Hormone konnten als zentrale Regulatoren des Energiestoffwechsels identifiziert werden. Sie regulieren homöostatische und hedonische Kreisläufe, welche das Essverhalten beeinflussen und eine adäquate Kalorienaufnahme zur Energiebedarfsdeckung gewährleisten. Die veränderte Hormonsekretion nach Gewichtsreduktion ist eine zentrale Ursache der Aktivierung von physiologischen Prozessen, welche eine neuerliche Gewichts- und Körperfettzunahme bewirken [4]. Hierbei kommt es zu kompensatorischen Änderungen im Hormonprofil mit einer reduzierten Sekretion von anorexigenen Hormonen wie „glucagon-like peptide 1“ (GLP-1), Peptide YY (PYY), Cholecystokinin (CCK), Amylin und Leptin sowie zu steigenden Spiegeln von orexigenen Hormonen wie Ghrelin und pankreatischem Polypeptid. Diese Anpassungen resultieren in einem vermindertem Sättigungsgefühl, vermehrtem Hungergefühl und einem gesteigertem Verlangen nach Nahrung [5–8].

Nach einer Gewichtsabnahme konnten im Vergleich zu einer normalgewichtigen Gruppe bei Menschen mit Adipositas niedrigere postprandiale Spiegel von GLP‑1, PYY und CCK bis zu einem Jahr nach der Intervention gefunden werden [9–11]. Das Ansprechen auf Veränderungen der gastrointestinalen Hormone im Zuge einer Kalorienrestriktion scheint variabel und somit mitbestimmend für das individuelle Risiko einer postinterventionellen Gewichtsabnahme zu sein [4].

### Metabolische Adaptierung

Der Grundumsatz (GU) beträgt rund 60–70 % des täglichen Gesamtenergiebedarfs und ist hauptsächlich von der Körperzusammensetzung, insbesondere der fettfreien Masse (FFM) abhängig. Eine Abnahme der FFM geht mit einer Reduktion des GU einher. Allerdings kommt es nach einer Gewichtsreduktion zu einer größeren Abnahme des GU als ausschließlich durch die Änderung der Körperzusammensetzung zu erklären wäre. Dieser Unterschied wird als metabolische Adaptierung (MA) bezeichnet und kann ein klinisch relevantes Ausmaß annehmen. Es wird angenommen, dass auch hormonelle Faktoren (Leptin, Schilddrüsenhormone, Insulin) für die Reaktion des Körpers auf eine Kalorienrestriktion über Beeinflussung der Herzfrequenz, des Blutdrucks und des sympathischen Nervensystems, verantwortlich sein dürften [12–15]. Es konnte gezeigt werden, dass die MA über einen längeren Zeitraum und auch nach einer Gewichtszunahme bestehen bleiben kann [15]. Individuelle Abweichungen in der MA könnten mitverantwortlich für Unterschiede bei der postinterventionellen Gewichtszunahme sein [16].

### Neurobiologische Vulnerabilität

Heißhunger und das Ansprechen auf Nahrungsreize nehmen insbesondere bei Menschen zu, die sich einer Kalorienrestriktion unterziehen. Dieser Effekt scheint bei Menschen mit Adipositas besonders ausgeprägt zu sein [17, 18]. Diese Vulnerabilität dürfte deutlichen individuellen Schwankungen unterliegen. Mittels funktioneller Magnetresonanztomographie wird versucht, prädiktive neurobiologische Marker im Belohnungssystem, die appetitanregende Kreisläufe aktivieren und so zu Hyperphagie und Gewichtszunahme führen, zu identifizieren [19]. Diesbezüglich steht die Forschung jedoch noch am Anfang.

## Beurteilung einer relevanten postinterventionellen Gewichtszunahme

Da der Energiebedarf direkt mit der Körpermasse in Verbindung steht, wird die Verwendung des Nadir-Gewichts als Referenz zur Beurteilung einer relevanten Gewichtszunahme empfohlen. Sie sollte pro Zeitintervall abgebildet werden [14]. Das Ausmaß der durchschnittlichen Gewichtszunahme pro Monat, ausgehend vom Nadir-Gewicht, kann folgendermaßen beurteilt werden:

- minimal: < 0,2 %,
- gering: 0,2 bis < 0,5 %,
- moderat: 0,5–1 %,
- rasch: > 1 %.

Eine rasche Gewichtszunahme von > 1 % pro Monat führt somit zu einer klinisch relevanten Gewichtsveränderung von > 5 % innerhalb von 6 Monaten. Um einer Gewichtszunahme zeitnah entgegenwirken zu können, empfiehlt es sich, mit dem Patienten am vorläufigen Interventionsende, ein „Warngewicht“, dass sich an einer durchschnittlichen geringen Gewichtszunahme (< 0,5 % pro Monat) orientiert, zu vereinbaren.

- *Beispiel:* Ein Patient wiegt nach der Intervention 100 kg (= Nadir-Gewicht). Ein Warngewicht von 5 kg, das wäre eine Gewichtszunahme von < 0,5 % vom Nadir-Gewicht über 12 Monate, wird vereinbart.

Bei Erreichen des Warngewichts sollte eine rasche Kontrolle am betreuenden Zentrum bzw. der Ordination erfolgen, um individuelle Maßnahmen zur Vermeidung einer weiteren Gewichtszunahme einzuleiten [2, 14].

## Maßnahmen zur Prävention von Gewichtszunahme

### Präinterventionelle Abklärung und Aufklärung

Eine sorgfältige präinterventionelle Abklärung zur Identifikation von Faktoren, welche eine postinterventionelle Gewichtszunahme begünstigen (Tab. 1), wird empfohlen. Zusätzlich sollten mit den PatientInnen die physiologischen Mechanismen einer Gewichtsreduktion bzw. -zunahme und die damit verbundenen Benefits und Risiken für die persönliche Gesundheit besprochen werden, um das Verständnis für die geplanten Maßnahmen zu stärken. Für die Gewichtsreduktion soll eine sinnvolle Geschwindigkeit und eine klare, realistische Zieldefinition (hinsichtlich Köpergewicht, Kleidergröße, Bewegungsziele etc.) vorgegeben werden [3, 20].

### Postinterventionelle Vereinbarungen und Monitoring

**Figure 1:**
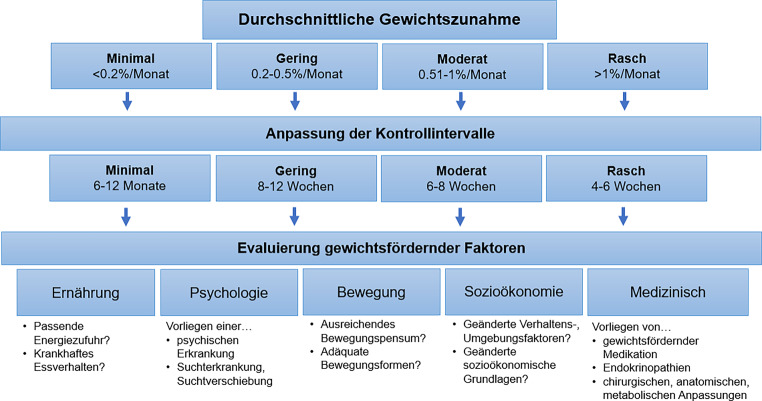

Postinterventionelle Vereinbarungen sollen regelmäßige Gewichtskontrollen (1- bis 2‑mal pro Woche) sowie ein individuelles Warngewicht (s. oben) umfassen [2]. Im ersten Jahr nach konservativer Gewichtsabnahme und bariatrischer Operation [1] sollen regelmäßige medizinische, diätologische, sportmedizinische und psychologische Kontrolltermine, zumindest alle 3 Monate, eingeplant werden (Tab. 2, Maßnahmen 2.1). Die Kontrollintervalle sollen abhängig vom Ausmaß der Gewichtszunahme angepasst werden (Abb. 1; [14]). Bei Erreichen des vereinbarten Warngewichts wird eine umgehende Wiedervorstellung im betreuenden Zentrum/in der Ordination empfohlen [2].

### Pharmakotherapie

Allfällige medikamentöse Therapie sollte v. a. hinsichtlich möglicher gewichtssteigernder Medikamente (Tab. 1, Faktoren 1.1) evaluiert werden [21]. Zusätzlich ist die Einleitung bzw. Anpassung einer antiadipösen Pharmakotherapie (GLP-1 Rezeptoragonisten, Naltrexon/Bupropion, Lipasehemmer) zu erwägen [1, 20–22].

## Management von PatientInnen mit Gewichtszunahme

Bei einer minimalen bis geringen Gewichtszunahme stehen die Evaluierung und Forcierung der Lebensstilmaßnahmen (Ernährung, Bewegung, Verhalten; Tab. 2, Maßnahmen 2.3–2.5) im Vordergrund. Bei den jeweiligen Kontrollterminen, deren Intervalle an die Geschwindigkeit der Gewichtszunahme angepasst werden sollten, empfiehlt sich die Erhebung von gewichtsfördernden Faktoren (Tab. 1). Bei moderater und rascher Gewichtszunahme sollte neben den Lebensstilmaßnahmen an eine Erweiterung der Therapie mit ggf. Einsatz einer Pharmakotherapie zur Gewichtsreduktion und/oder chirurgischen Maßnahmen gedacht werden [1, 20–23].

### Rehabilitationsaufenthalt mit Stoffwechselschwerpunkt

Eine stationäre Rehabilitation empfiehlt sich, um Abstand von den Alltagsroutinen zu bekommen und die biopsychosozialen Umstände der neuerlichen Gewichtszunahme hinterfragen, verstehen und verändern zu können. Daran anschließend sollte dem/der PatientIn eine längerfristige multiprofessionelle Begleitung im Alltag (medizinisch, psychologisch, diätologisch) angeraten werden.
